# Using an unbiased symbolic movement representation to characterize Parkinson’s disease states

**DOI:** 10.1038/s41598-020-64181-3

**Published:** 2020-04-30

**Authors:** Avner Abrami, Stephen Heisig, Vesper Ramos, Kevin C. Thomas, Bryan K. Ho, Vittorio Caggiano

**Affiliations:** 1 IBM Research - Healthcare and Life Sciences - 1101 Kitchawan Rd, Yorktown Heights, NY 10598 USA; 2 Digital Medicine and the Pfizer Innovation Research Lab, Pfizer, 610 Main Street, Cambridge, MA 02139 USA; 3 Laboratory for Human Neurobiology, Spivack Center for Clinical and Translational Neuroscience, 650 Albany Street, X-140, Boston, MA 02118 USA; 4 Department of Neurology Tufts Medical Center 800 Washington Street, Box 314, Boston, MA 02111-1800 USA

**Keywords:** Motor control, Parkinson's disease

## Abstract

Unconstrained human movement can be broken down into a series of stereotyped motifs or ‘syllables’ in an unsupervised fashion. Sequences of these syllables can be represented by symbols and characterized by a statistical grammar which varies with external situational context and internal neurological state. By first constructing a Markov chain from the transitions between these syllables then calculating the stationary distribution of this chain, we estimate the overall severity of Parkinson’s symptoms by capturing the increasingly disorganized transitions between syllables as motor impairment increases. Comparing stationary distributions of movement syllables has several advantages over traditional neurologist administered in-clinic assessments. This technique can be used on unconstrained at-home behavior as well as scripted in-clinic exercises, it avoids differences across human evaluators, and can be used continuously without requiring scripted tasks be performed. We demonstrate the effectiveness of this technique using movement data captured with commercially available wrist worn sensors in 35 participants with Parkinson’s disease in-clinic and 25 participants monitored at home.

## Introduction

Parkinson’s disease (PD) is a neurodegenerative disease of incompletely understood etiology with no diagnostic test that affects over one million people in the US^1^. Neuropathologically, it causes degeneration of dopamine secreting neurons in the substantia nigra region of the basal ganglia^2^. The result is less dopamine produced by this area of the motor system. Dopamine is a neurotransmitter which facilitates and modulates the activity of neurons. The clinical manifestations of this degeneration are observed in the dysregulation of motor control leading to the hallmarks of Parkinson’s disease: tremor, bradykinesia, rigidity, gait and balance problems, and dysdiadochokinesia.

The current gold standard instrument for assessing PD is the clinician administered Movement Disorder Society Unified Parkinson’s Disease Rating Scale (MDS-UPDRS) test^3^. The examination occurs in-clinic and consists of three main parts. Part three (UPDRS-III) is dedicated to clinician impressions of motor signs observed in the patient. In this section the clinician asks the patient to perform tasks such as walking, finger tapping, and foot stomping which are designed to evince motor signs of the disease. The clinician then subjectively rates the amplitude, frequency, and quality of these tasks on a scale of 0 to 4. Typically, this examination is given once or twice a year and is confounded by the stress of travelling to the clinic, the state of the patient with respect to their medication cycle, the so-called “white coat effect” (i.e. stress of a clinic visit), and clinician training and experience. For example, senior movement disorder specialists have been found to assign lower scores than residents or younger colleagues^4^.

There is currently no approved treatment which changes the progression rate of Parkinson’s disease. The gold standard treatment for Parkinson’s disease is dopamine replacement therapy (DRT) which compensates for the lack of dopamine produced endogenously. Prescribing the appropriate amount of drug is important since not enough leads to increased risk of falling and poor symptom control. Too much drug leads to faster habituation and potentially disabling dyskinesias. The consequences of a poorly tuned prescription, the challenges and costly nature of neurologist visits, and ultimately the burden to patients motivates the development of an evaluation technique appropriate for in-home use that does not require the performance of scripted tasks.

Wearable technologies^5^ allow continuous monitoring of movement from different parts of the body. They provide data for potential biomarkers of disease state and progression and to assess the effect of treatment inside and outside the clinical environment ^6,7^. Attempts have been made at home self-monitoring through technologies including smartphones (e.g^8–10^.) and motion sensors attached to different body parts (e.g. lumbar sensors^11,12^, wrist sensors or on shoes^13,14^). For a comprehensive summary see Del Din *et al*.^15^ and Monje *et al*.^7^. Many of these methods require an instrumented setting with scripted tasks – potentially affected by cognitive deficits associated with the disease – or they focus only on a single impairment^10,16–19^ e.g. gait disturbances, postural imbalance, tremor, bradykinesia, rather than overall motor symptoms. While these are effective at capturing specific motor aspects of the disease, they are not able to capture the overall disease state, providing only low^20,21^ or moderate regression accuracy with respect to overall motor impairment^22^. There is a need for a method that continually extracts information about motor disease state from patients in an objective manner, without interfering with everyday life or requiring active participation beyond charging the device.

The theoretical perspective of animal movement as a continuous sequence of stereotyped motifs governed by a syntax of action akin to syllables in language is historic^23^. Lashley explicitly suggested that language, music, and complex chains of movements shared a hierarchical structure resulting from use of a common motor planning apparatus. One of the biggest challenges in monitoring the quality of movements in everyday life is the wide variety of modes of locomotor and manual activities that healthy individuals exhibit interacting with the environment. Previous work has shown that the mammalian central nervous system uses a reduced set of movement patterns^24–26^ such that movement kinematics can be explained in a compressed space of dynamic primitives^27^. Expressed sequentially, these movements result in naturalistic behaviours^28,29^. Unsupervised and unbiased methods have been shown to be able to extract those movement components both in animals^24,29^ and in humans in constrained settings^30^. These movements have also been called primitives, or behavioral motifs, or syllables^28^. In this paper, we will use a symbolic representation of movement syllables. Since the order of the sequence of symbols is affected by injuries and diseases involving the motor system^29^, they provide a window into neurological state. In particular, PD is known to affect the sequence of movements in addition to the movement itself^31,32^. Nevertheless, the extraction of syllables as an ordered sequence in unconstrained every day movements has never been performed. The hypothesis of disorganized sequences of syllables as a result of PD state has never been addressed using human movements in everyday activities.

Our approach to estimating changes in PD state is based on discovering basic motion syllables measured at the wrist using a wearable accelerometer with unbiased techniques. A set of wrist movement syllables is defined and detected in continuous data using a k-means clustering model. The stationary distribution of syllables represents the statistical grammar of movement during that interval and we observe the increasing dysregulation of movement as the neuropathology of the disease progresses. We will refer to the stationary distribution of syllables as the symbolic movement representation (SMR). In this paper we apply our approach to data from three studies as part of the Bluesky project^33^ that collected data from people diagnosed with PD undergoing the standard neurological exam, healthy participants undergoing the same protocol and people with PD in unconstrained behavior at home.

## Methods

### In-clinic participants

For the in-clinic studies^33^, participants with idiopathic PD were recruited, and the protocol was run at Tufts Medical Center, Boston, Massachusetts. The study protocol was approved by the Tufts Health Sciences Campus Institutional Review Board, IRB # 12371. All participants were over 18 years of age and gave written, informed consent prior to the start of the study. The protocol was carried out in accordance with the relevant guidelines and regulations documented in the IRB submission. Inclusion criteria included response to DRT, ability to recognize ON and OFF states, and an assessment of stage 3 or lower on the Hoehn and Yahr scale. Exclusion criteria were a current history of neurological disease besides PD, psychiatric illness that would interfere with participation, alcohol consumption exceeding 7 drinks per week for females and 24 drinks per week for males and any implanted device such as a cardiac pacemaker, electronic pump, or deep brain stimulation device.

Healthy participants were recruited, and the protocol was run at IBM and Pfizer sites. The study protocol was approved by the Schulman Independent Institutional Review Board IRB # 201500837. All participants were over 18 years of age and gave their written, informed consent prior to the start of the study. The protocol was carried out in accordance with the relevant guidelines and regulations documented in the IRB submission.

For this paper, we evaluated data from the first 45 healthy participants to enroll in the study (HP, age average 44 + /− 13 years old) and 35 participants (age average 68 + /− 8 years old) diagnosed with PD (see^34^ for the complete study design). Each participant performed up to 16 tasks involving walking and hand-movements or hand-object interactions as part of their scripted activities (see Table 1). These were designed to simulate activities of daily living (ADLs). Participants wore Opal devices (APDM Wearable Technologies) on both wrists, feet, chest and lumbar. These inertial measurement units (IMUs) captured movement across 9-axes (3-axis accelerometer, 3-axis gyroscope, and 3-axis magnetometers) at 128 Hz. In this study only data from the 3-axis accelerometers on each wrist was used. We used accelerometer information only because accelerometers are available in many commercial products (integrated in sport watches or step monitors) and they use less power than gyroscopes – which would allow more time between charges. PD participants were assessed by a neurologist specializing in movement disorders (Dr. Ho) before performing the tasks with a standard UPDRS-III exam. We added bradykinesia, tremor and gait subscores (see Scores Definition below) to calculate the total UPDRS-III score. Data was collected during two visits for each PD participant. In one visit, the participant was tested after taking their usual DRT medication (ON-state), in the other after the effect of the medication had worn off (OFF-state). The ability to discriminate ON from OFF states was a prerequisite for participation in the study. ON and OFF status at the start of each session was confirmed by the participants and verified by the neurologist. Healthy participants screened for neurological problems performed both visits in their normal state. At each visit all participants performed a set of scripted tasks (see Table 1). More details about the study can be found in^33^.

**Table 1:** Duration of the Activities of the Daily Living (ADL) for Healthy (HP) and Parkinson’s Disease (PD) participants (see^33^ for more details).

	All Healthy Participants (HP) N = 45	Eldest HP N = 8	Parkinson’s Participants N = 35 (tested both ON and OFF states)
Age (years old)	44 ± 13	61 ± 5	68 ± 8
Gender (Male vs Female)	20 vs 25	2 vs 6	23 vs 12
Handedness (Right vs Left)	41 vs 4	8 vs 0	29 vs 6
**Duration Session in seconds**			
BookCarrying	18.91 ± 3.88	18.38 ± 2.33	26.84 ± 12.94
BottleShaking	13.04 ± 2.13	13.25 ± 1.75	19.11 ± 9.0
CoatButtoning	47.87 ± 8.37	48.38 ± 8.18	103.09 ± 61.75
CursiveWriting	55.96 ± 10.71	51.75 ± 5.44	69.74 ± 29.06
Door	12.04 ± 2.56	12.0 ± 2.56	9.49 ± 3.83
FoldingPaper	25.36 ± 5.39	23.62 ± 4.27	38.53 ± 27.24
GaitWalk10m	31.97 ± 28.81	30.88 ± 23.26	120.94 ± 5.27
GaitWalk2.5 m	23.17 ± 3.86	22.94 ± 4.85	31.54 ± 2.49
Jewelry	13.49 ± 4.0	12.5 ± 3.02	15.13 ± 6.81
PouringDrinking	21.78 ± 4.39	22.0 ± 2.07	27.04 ± 19.29
Remote	12.29 ± 2.23	11.25 ± 1.75	13.86 ± 4.9
SentenceWriting	18.71 ± 3.12	18.12 ± 2.53	53.66 ± 18.79
ShoeTying	29.51 ± 16.71	26.25 ± 7.36	49.99 ± 36.19
SpoonEating	18.44 ± 3.37	19.38 ± 3.25	23.81 ± 14.92
SuitcaseCarrying	67.6 ± 26.8	61.5 ± 27.6	119.33 ± 15.3
Zipping	36.73 ± 8.29	38.12 ± 5.28	62.67 ± 36.24

All values are mean ± standard deviation.

### At-home participants

For the at-home studies, participants were recruited and the protocol was run at Boston University School of Medicine. The study experimental protocol was approved by the Boston University School of Medicine Institutional Review Board (NCT0324738). All participants were over 18 years of age and gave their written, informed consent prior to the start of the study. All of the methods were carried out in accordance with the relevant guidelines and regulations documented in the IRB submission. In this study, we evaluated data from 26 participants (age average 67 + /− 8 years old) diagnosed with PD (see^33^, total UPDRS-III score 25 + /− 13). Each participant wore a GeneActive device (Activinsights) equipped with a 3-axis accelerometer sampling at 250 Hz on the non-dominant wrist continually for 2 periods of 3 days each. The second period was separated from the first by 30 days. Participants were assessed by a movement disorder specialist before the data collection period with a standard UPDRS-III exam in the ON-state. More details about the study can be found in^33^. Post-acquisition, one participant was excluded because their data was only partially available due to a device failure. Another participant’s data showed abnormal behavior in the quantile-quantile plot and failed the Cook test with a value of 0.47 (the standard threshold for exclusion is any value above 0.16). Therefore, we excluded this participant.

### Data preprocessing

Signals from wrist sensors were first down-sampled to 25 Hz and then band-pass filtered between 0.2 and 3 Hz (see Supplementary Figure 1) with a Butterworth filter (order 3). The y-axis for the right wrist was inverted so that both hands had the same relative trajectory respect to the body. A sample was defined as a multidimensional (x, y, and z axes) time series of accelerometer recordings contained in a 1 second window. Each window of 1 second at 25 Hz consisted of an array of 75 points (a concatenation of 1 second of 25 points for 3 axes) for 3-axial acceleration from one wrist. Windows were incremented by a step of 0.24 seconds (an increment of 0.24 * 25 = 6 points) so that any single point appeared in four windows.

#### Tokenization of movement data

An ADL is then captured as a sequence of movement syllables ${W}_{i}^{t}\in \Omega$:

$$AD{L}_{i}=({W}_{i}^{{t}_{1}},{W}_{i}^{{t}_{2}},\mathrm{.}.,{W}_{i}^{{t}_{n}})$$

with

$${t}_{1} < {t}_{2} < \ldots  < {t}_{n}$$

Where $\Omega$ refers to the vocabulary of movement syllables, *t* is the starting time of the window and *i* refers to a defined ADL. A clinical visit is made of a collection of ADLs, i.e. a collection of symbolic sequences. We chose a vocabulary with 24 syllables based on the elbow method (see Supplementary Figure 2). Nevertheless, choosing a vocabulary size between 20 and 50 syllables did not significantly affect the results and conclusions of this manuscript.

### Embedding movements into a sequence of syllables

We used a Markov Chain (MC) to characterize our symbolic sequences. Each sequence provided transitions between syllables that are used to estimate the MC. Transition rates between syllables were estimated using maximum likelihood:

$${P}_{(a,b)}=\sum _{b\in \Omega }\frac{{N}_{(a,b)}}{{N}_{a}}$$

Where *P*_*(a,b)*_ is the transition rate between *a* and b, *N*_*a,b*_ is the number of times *a* is followed by *b*, *N*_*a*_ is the total number of times *a* occurs in the session.

Unseen transitions were ‘smoothed’ by a small value (1e^−10^) ensuring strictly positive transition rates for the transition matrix, which makes the MC irreducible. An irreducible discrete time MC over a finite state space has a unique stationary distribution, which constitutes the SMR.

#### A distance metric to assess motor behavior

The estimator $\widehat{SMR}$ is obtained by solving the following linear system:

$$\widehat{SMR}=P\widehat{SMR}$$

The $\widehat{SMR}$ represents the frequency of each movement word over a long-time period and embodies the motor state of the participant during that session.

We used the same total variation distance - in a finite space it corresponds to the ${L}_{1}$ norm - to compare the SMR between sessions:

$${D}_{i,j}=d(SM{R}_{i},SM{R}_{j})={\Vert SM{R}_{i}-SM{R}_{j}\Vert }_{1}$$

for all

$$i,j\in \Psi $$

where Ψ refers to the set of sessions. Those distances are stored in *D*, which is the dissimilarity matrix between each session. $D$ is symmetric and its diagonal values are zero, meaning that zero is the measure of dissimilarity between a session and itself. Conversely, a greater distance between SMRs (reflecting higher differences in motor behavior) will be indicated by an increasing ${L}_{1}\,$distance.

In order to quantify each session with respect to its global UPDRS-III score, we computed each session’s position in a 3D embedded space where distances between SMRs are preserved, using the multi-dimensional scaling algorithm (MDS)^35^.

#### Scores used from the Part III of the Movement Disorder Society Unified Parkinson’s Disease Rating Scale (UPDRS-III)

##### Bradykinesia score:

 Pronation Supination Right, Pronation Supination Left, Toe Tapping Right, Toe Tapping Left, Leg Right, Leg Left, Tapping Right, Tapping Left, Hand Movement Right, Hand Movement Left, Rigidity Neck, Rigidity Upper Right, Rigidity Upper Left, Rigidity Lower Right, Rigidity Lower Left.

##### Tremor Score:

Postural Tremor Right, Postural Tremor Left, Kinetic Tremor Right, Kinetic tremor Left, Rest Tremor Amplitude Upper Right, Rest Tremor Amplitude Upper Left, Rest Tremor Amplitude Lower Right, Rest Tremor Amplitude Lower Left, Amplitude Lip, Rest tremor constancy.

##### Postural Instability and Gait Disorder (PIGD):

Arising from chair, Posture, Gait Score, Freeze Score, Postural stability.

##### Total UPDRS-III:

Bradykinesia Score, Tremor Score, PIGD, Facial Expression Score, Speech Score.

## Results

### Definition of a symbolic movement representation from continuous time series data

**Figure 1:**
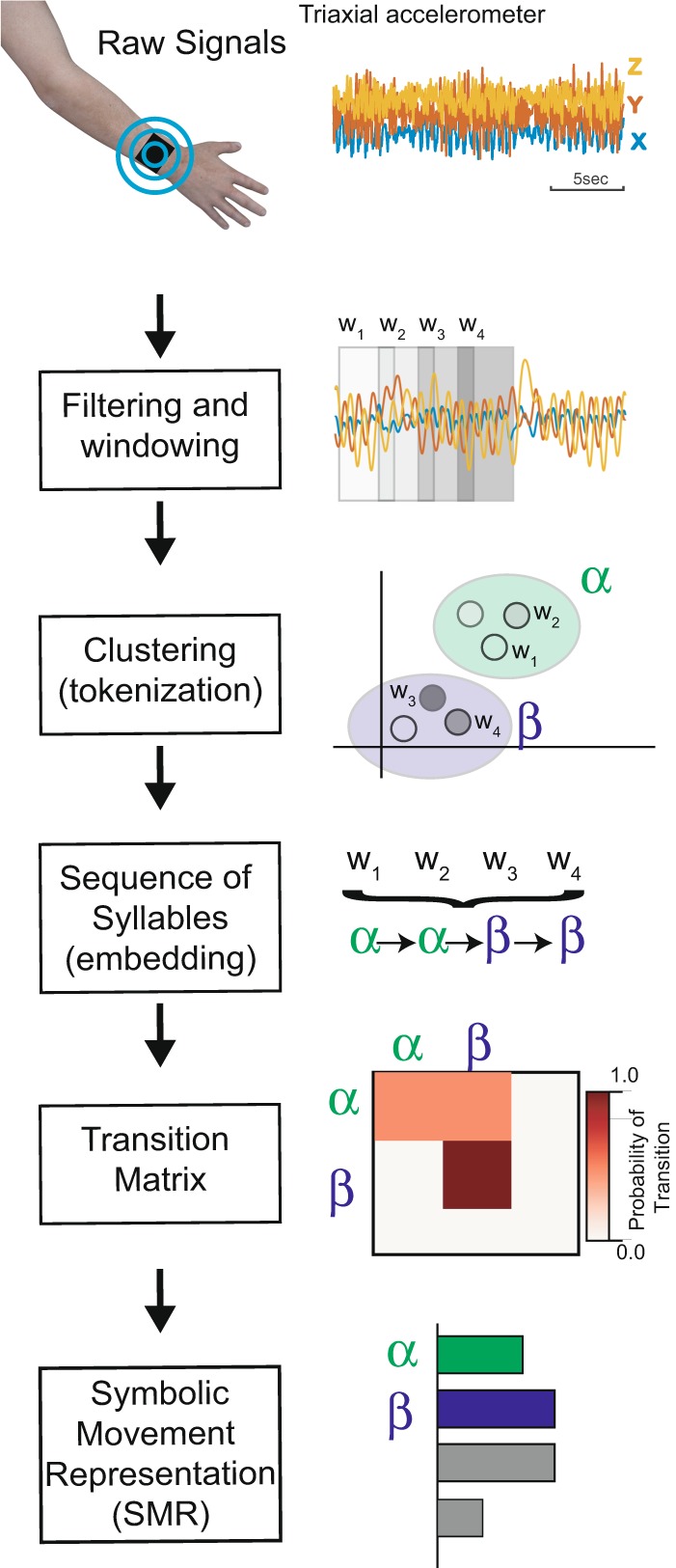
Transformation of movement time series into a Symbolic Movement Representation. Schematics of the pipeline: raw data from wrist sensor is first subsampled, band-passed filtered, and taken in windows of 1 second with overlap of 240 ms. Each window is mapped to a discrete syllables (Clustering-Tokenization). By associating each window of activity to a cluster, the continuous signal is transformed into a discrete sequence of syllables (Sequence-Embedding). The sequence can be seen as a discrete markov chain and each action can be then represented by a Transition Matrix from a Markov-Chain. An SMR (see main text) is estimated as the Symbolic Distribution estimator of the transition matrix obtained performing several actions.

**Figure 2:**
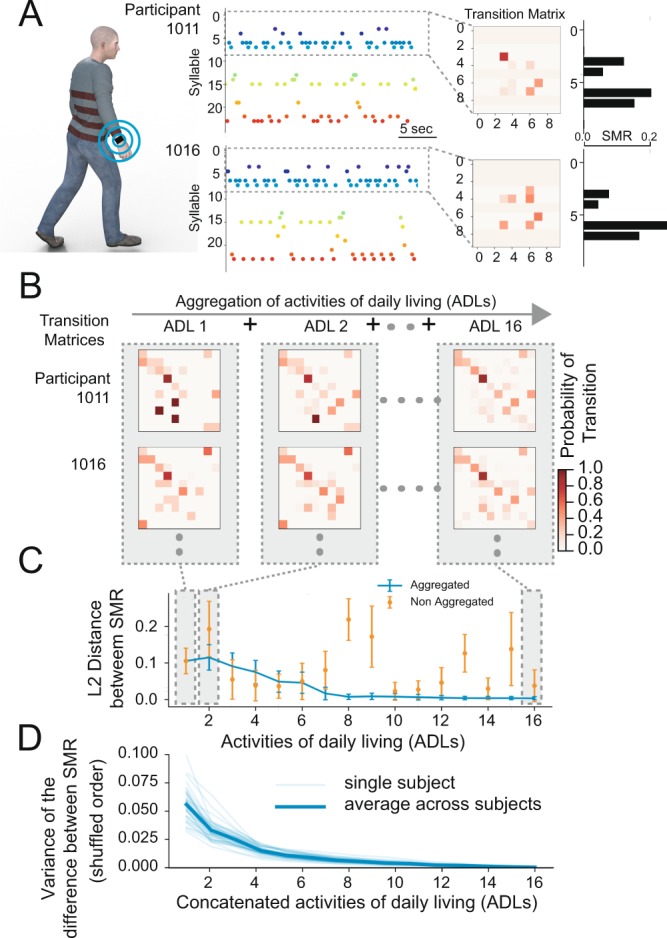
Symbolic Movement Representation in healthy participants. (**A**) Examples of sequences of syllable over time during a walking task. In the inset the transition matrix (only the first 10 syllable are shown for illustrative purposes) and their corresponding SMR for the first 10 syllable. Note that the index of the syllable starts from 0. (**B**) Examples of transition matrices from different participants (rows – only the first 10 syllable are shown for illustrative purposes). When several actions are concatencated, the sequence of actions converges to a similar transition matrix (right column). (**C**) Distance between pairwise SMR when actions are considered in isolation (orange) or aggregated (blue). (**F**) Variance of SMRs first PCA as a function of concatenated activities. For each participant (light blue curve), a shuffled order of ADLs was generated for 50 times and the SMR for the concatenated ADLs was computed. Then, the average of the variance of the first PCA of the SMR was computed. Thick blue line indicates the average across participants. For a given ADL number (1 to 16), SMRs are simulated (corresponding to different sets of ADLs) and dimensionally reduced to their first PCA. As the number of concatenated ADLs increases, the variance of their PCA components decreases showing the stability of the SMR estimator.

Our first objective was to discover a set of movement syllables independent of the specific activities performed that captured the overall characteristics of wrist movement. We then described continuous movement as a sequence of movement syllables represented by symbols. The Symbolic Movement Representation (SMR), defined as the stationary transition distribution between syllables captures the quality of movements while maintaining task independence. For this purpose, we used continuous accelerometer data recorded from wrist sensors^33,36^ worn on the non-dominant hand of healthy participants performing scripted actions mimicking ADLs, e.g. writing, walking, eating (see Table 1). Those activities were performed with different movement features e.g. peak acceleration and peak frequency (see Supplementary Figure 1) and covered most of the movements produced in a home environment. Continuous time series data was analyzed in windows (1 second with overlap of 0.24 seconds, Fig. 1 – Filtering and windowing) and mapped into syllables (N = 24) using the K-Means clustering algorithm (Fig. 1 – Clustering). This latter step enabled us to represent the continuous time series as a sequence of syllables (Fig. 1A–Sequence and Fig. 2A, see also Supplementary Figure 3). This sequence was then modeled as a discrete time Markov Chain (MC) in which each syllable was a state (Figs. 1–2A – Transition Matrix): each action is characterized by a sequence of syllables (see Figs. 1A–2B, Supplementary Figure 4).

Although humans move in slightly different ways, the transitions between states show similarities across participants. For example, Fig. 2A shows a sequence of syllables over time for two participants and their correspondent transition matrices.

Our hypothesis was that during daily life, people perform common sequences of movements. These are part of our learned motor repertoire with common subsequences shared across different actions. We demonstrated this by looking at transitions between syllables both for one single action (each ADL independently) and by concatenating different actions (multiple ADLs). Indeed, after aggregating enough ADLs, the transition matrix (TM) of the Markov Chain for each participant converged to a stereotypical distribution (last column of Fig. 2B). Another way to look at this phenomenon is to compute the Stationary Distribution of the Markov Chain (see Methods), henceforth defined as the Symbolic Movement Representation (SMR, Figs. 1–2A) and to measure the distance between the SMRs among participants. Indeed, the SMR did not change between participants when more than 8 actions are concatenated (see blue line Fig. 2C). Conversely, when ADLs are not concatenated (orange line Fig. 2C) there is a greater variability between actions of different participants (see Supplementary Figure 5 for how the projection of the SMR on the first 2 principal components differentiates a single action in isolation - orange dots - or aggregating several actions – blue dots). We found that with more than 9 ADLs each participant reached a stable SMR estimator independently of the precise order in which the actions were concatenated (see Fig. 2C,D). The transformation of continuous movements into an SMR allows us to describe the most common motor behavior across participants and actions.

### Symbolic movement representation applied to actions in people with PD during scripted activities of daily living

**Figure 3:**
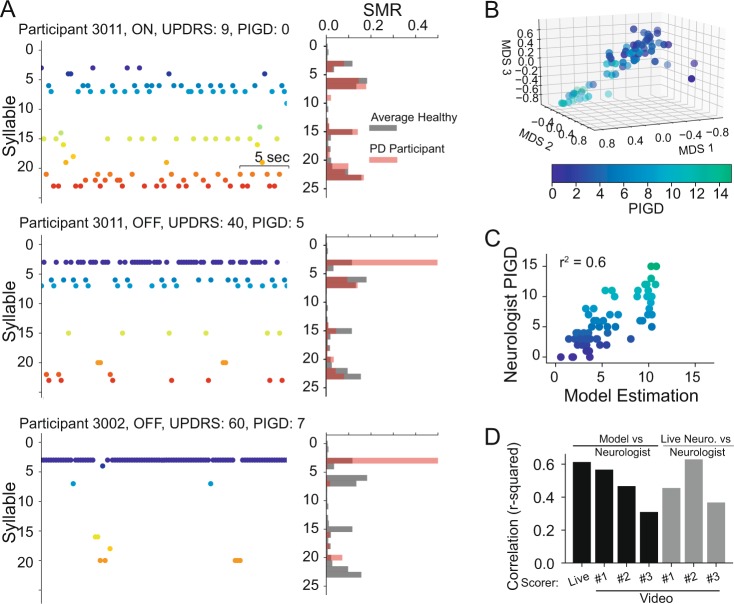
Sequence of movements in the Walking task. (**A**) Examples of Sequences of movements during 24 seconds of the walking ADL for two PD participants. One PD participant is shown in two visits with two different UPDRS-III scores. Note that the index of the syllable starts from 0. (**B**) Multi-Dimensional Scaling of the distances between SMR of all PD participants. The correspondent PIGD value for each participant is color coded. (**C**) Estimation of the PIGD based on a linear model trained on the 3 components of the MDS. (**D**) Model estimation (black bars) measured as r-squared correlation when the MDS distances are regressed against neurologist examining the participant in person (Live) or through video clips i.e. Video Scorers. Also, the same model was built to predict the Live scorer based on the Video Scorers (gray bars).

Once we established that individual motor behavior can be expressed in terms of an SMR, we used the same approach to analyze movements in 35 participants with PD (see^33^). In this case, we built the SMR using the symbolic representation learned from the whole population of healthy participants and, as in the previous exercise, we generated sequences of actions and their SMR from continuous activity in windows (1 second with 0.24 seconds overlap) using the same syllables learned previously from healthy participants. Furthermore, we considered each PD participant twice, independent of the reported medication state (ON or OFF), for a total of 70 data points and compared them against eight eldest healthy participants (HP, see Table 1). We used the healthy actions as contrast because their actions provided a more complete representation of movements. Indeed, the variance of movements (as reported in the first 2 PCAs of the raw movements) in PD participants was significantly smaller than for healthy participants (Levene-test for equal variances p <0.05, variance healthy vs PD, PC1 25.8 vs 13.2, PC2 25.6 vs 12.2). See also Fig. 3A for the effect of the disease on the variability of actions in PD as function of the disease severity.

The analysis of movements in terms of transitions between syllables shows a remarkable feature of disease progression. By considering only the sequences during gait movements, we observed different sequences in different PD participants (see Fig. 3A). We applied Multi-Dimensional Scaling (MDS - Methods) to the SMR distances (see in Fig. 3B) to enhance relative positions such that the pairwise dissimilarities (distances) between participants were preserved. The resulting scaled distances were highly correlated (r^2^ = 0.6, p <0.05, linear regression model applied to three MDS axes, Fig. 3C) with the cumulative neurologist score for posture and gait impairment (Live Scorer in Fig. 3D). Similar correlations were also obtained when the model was compared against the UPDRS-III scores assigned by the neurologist analyzing the video clips of the MDS-UPDRS (defined as Video Scorers, see Methods). Our estimations were in the same range as the interrater variability (see gray bars, Fig. 3D). This result, although indicative of a robust assessment of the motor impairment relied on the construction of the SMR on a specific walking task and cannot be generalized to other hand motor behavior.

**Figure 4:**
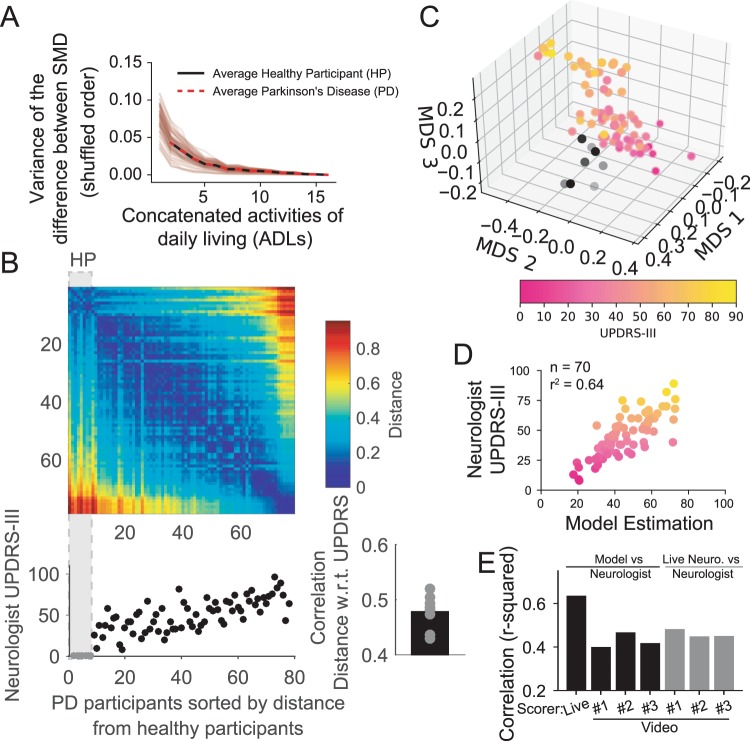
Relationship of SMR and degree of motor impairment. (**A**) In red, Parkinson’s disease (PD) participants. In black, eldest healthy participants (HP). (**B**) On the top, a distance matrix showing the pairwise L1 distances between each SMR both for healthy (first 8 values - HP) and PD participants. Columns are sorted based on the distance from the averaged HP distribution. On the bottom, total UPRDS-III score of each PD participant sorted in the distance matrix (bottom left) and the correlation between of the L-1 distance of PD participants with respect to each HP and their UPDRS-III values. On average, sorting PD participants according to their distance from any HP produced an average correlation of 0.47. (**C**) MDS of the distances of B. The correspondent total UPRDS-III value for each participant is color coded. (**D**) Estimation of the UPDRS-III based on a linear model trained on the 3 components of the MDS. (**E**) Model estimation (black bars) measured as r-squared correlation when the MDS distances are regressed against neurologist examining the participant in person (Live) or through video clips i.e. Video Scorers. Also, the same models were built to predict the Live scorer based on the Video Scorers (gray bars).

In order to generalize the proposed method across different ADLs, similar to the previous analysis for healthy participants (see Fig. 2D), we built an estimator of the SMR by concatenating ADL data. A stable estimator for this new dataset was reached after aggregating more than 9 ADLs (see Fig. 4A). This result indicates that movements in people with PD over the course of different motor activities also converge to a stable SMR estimator.

Next, we examined how the SMR varied with the overall severity of motor impairments. First, we computed a dissimilarity matrix using the pairwise distance between the SMR of every participant in our datasets of the eldest healthy participants and PD participants. The dissimilarity matrix was then ordered by the distance of PD participants against the averaged healthy participants’ SMRs. As seen in the color-coded matrix of Fig. 3B, participants with similar distances are represented by blue regions of low dissimilarity (e.g. among healthy participants in the upper left corner). Indeed, healthy participants have very small intra- participants differences (i.e. 0.18 + /− 0.04) while, PD participants showed higher differences and variability (0.29 + /− 0.15, p <0.005 t-test – mean – and p <0.005 F-test – variance – between PD participants and healthy participants).

When the total impairment assessed by the neurologist (total MDS-UPDRS-III) was sorted based on the distance between PD and healthy (we assumed that the healthy participants had a score 0 although they were not rated by a neurologist), a greater distance from healthy participants was related to a higher impairment score. We quantified this observation in two ways: first, we computed the distances between all PD participants and each healthy participant (8 combinations) and correlated those distances against their respective total UPDRS-III score. All cases were highly correlated with the UPDRS-III score (see inset on the right of Fig. 3B, average r-squared> 0.43, p <0.05). Second, we applied Multi-Dimensional Scaling (MDS - Methods) to the SMR dissimilarity matrix to create a 3D embedding of the different motor behaviors (similarly as done previously for gait, also see Methods). In this embedding (see Fig. 3C) the oldest healthy individuals (in black/gray – see Supplementary Figure 6 for the whole population of healthy vs PD) are separable from the rest of the PD population (color-coded according to their UPDRS-III score). Most importantly, MDS values of participants with PD showed a clear pattern of separation from healthy participants proportional to their impairment (i.e. greater UPDRS-III scores were associated with greater distances from healthy participants). As shown in Fig. 4D, based on a linear regression model applied to the MDS values, we were able to estimate the UPDRS-III score with high accuracy (p <0.05, r-squared = 0.64 - multivariate linear regression of three MDS axes against UPDRS-III). Those results were minimally dependent on the specific choice of parameters (e.g. window size, number of clusters, or the position of the sensor, see Supplementary Figure 7). It is worth noting that the model built on the MDS space was better correlated with the UPDRS-III values when we used the assessment of motor impairment done by a neurologist analyzing the participant in person (i.e. Live Scorer, see Fig. 4E). When the model was built with UPDRS-III scores assigned by a neurologist analyzing the participant via video of the UPDRS-III tasks (defined as Video Scorers, see Methods), performance decreased to values below 0.5 (see Fig. 3E). Those values were still comparable to the interrater variability (see gray the bars Fig. 4E).

Finally, we tested our method at capturing specific aspects of the neurologist motor assessment as a function of the sensor placement. We examined wrist data from the dominant vs non-dominant hand and most affected vs least-affected hand. We created linear models to estimate both the total UPDRS-III score and subscores rating bradykinesia, tremor and postural or gait stability (PIGD). As indicated in Supplementary Figure 8, our model was well correlated with both the total UPDRS-III score (see for a parameter analysis Supplementary Figure 7), Bradykinesia score, and PIGD. This relationship was expected because those subscores are also highly related to the total UPDRS-III (see the first row of Supplementary Figure 8A). It is not a surprise that we had a very low tremor score correlation since our model excluded tremor related frequencies. Finally, we tested the effect of sensor position on capturing gait specific impairments when the model was built on data from the walking task (model shown in Fig. 3 and Supplementary Figure 8B). In this case, the best relationship was obtained with the subscore assigned to describe impairment of gait and posture (PIGD). These results suggest that the SMR applied to movements in people with PD captured their overall motor impairment during the execution of scripted activities independently of sensor position.

One of the factors that might affect the results is the age of the participants. In order to test the effect of age, we computed the MDS including all healthy and PD participants. We did not find any correlation of the MDS with age (p > 0.05) in healthy participants. It is worth mentioning that we found a low nevertheless significant correlation of the MDS with age in PD patients (r-squared = 0.19). This indicates a very little contribution of age on the overall correlation. Still, age usually correlates with overall PD severity. In our case, age weakly correlated with overall UPDRS score (r-squared = 0.23).

### Monitoring people with PD in everyday life

To test our method in an at-home setting, continuous data from 25 PD participants for six days was collected^33^. Collection was continuous in two sessions of three days each separated by 30 days. Participants wore a GeneActiv wrist device on the non-dominant hand day and night. Each participant was scored at the beginning and the end of the data collection period by the same neurologist with the participant in the ON state. Because of the variability between UPDRS-III scores at the endpoints, we used the average value of the UPDRS-III scores during the two visits for each participant as a reference.

Continuous data in this new dataset was preprocessed in the same fashion as for the scripted ADLs. In this case, we trained the clustering algorithm on the healthiest PD participant (i.e. the only participant with a UPDRS-III score less than 6) and we applied this model to the remaining participants.

Each day was divided into 30 minute-windows for which the SMR was estimated and mapped to a 3D MDS embedding space.

**Figure 5:**
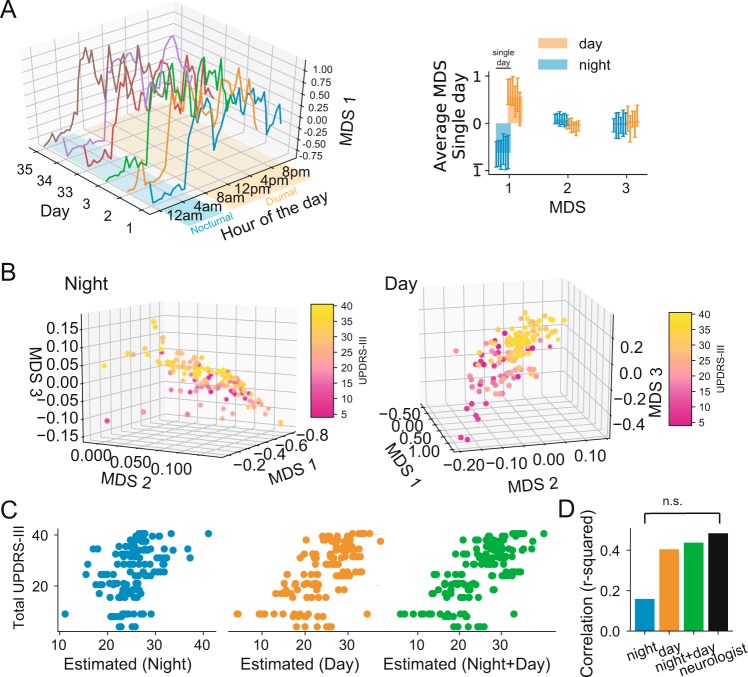
Validation of the data in the wild. (**A**) Example of the first (left) MDS projection of continuous data during the day across 6 different days for one participant and averages (right) of the 3 MDS projection during day and night (see text). (**B**) Representation of all participants in the MDS space for night (left) and day (right) activity. (**C**) Linear model regression based of night activity (left – blue), day activity (center – orange) and combining day and night activities (right – green) against the neurologist score. (**D**) Summary of the regression correlation values.

Inspection of the MDS embedding over time clearly revealed two phases: a diurnal phase and a nocturnal phase. We then restricted movements during nocturnal activity to 12 am to 6 am and diurnal activity to 8 am to 8 pm (see Fig. 5A). When we compared both the diurnal and nocturnal activity between participants a clear relationship with the degree of impairment emerged (see Fig. 5B). As shown in Fig. 5C, both diurnal and nocturnal activities were well correlated with the average UPDRS-III score assigned to each participant. Figure 5D summarizes the results of model estimation using only nocturnal, only diurnal or both activities to build a regression model. When we combine both activities, the estimation of the model shows roughly the same accuracy as the neurologist variability (p > 0.05, Steiger-test, neurologist indicated as the black bar in Fig. 5D).

## Discussion and Conclusion

In this paper, we demonstrated an unsupervised technique to generate objective measurements of movement quality during in-clinic and at-home activities. By transforming continuous signals from wearables into a statistical distribution of movement syllables (the symbolic movement representation) we captured the increasing disorder in motion between healthy and disease state. This statistical representation also correlated with increased motor impairment among people with PD as quantified by their MDS-UPDRS scores. This correlation was highly accurate (r-squared up to 0.64) at predicting neurologist scores when scripted tasks were performed in-clinical and in the naturalistic at-home setting.

Our unsupervised method for generating movement statistics can be compared to the analysis of power law exponents in actigraphy^37,38^ and micro-movements^39^. The critical difference is that our model uses specific movement syllables and their sequence distribution rather than absolute information related to a specific physical activity or variability in micro-movements. Also, in contrast to actigraphy-derived descriptors of movements requiring large amounts of data to build their statistics, our approach needed only a few actions (less than 2 minutes) to create stable descriptors. In this aspect, it is better suited to a more granular continuous assessment (see Fig. 5A) to detect the effects of therapy over the course of the day.

Purposeful and meaningful behaviors are built on sequences of movements^23^ which are organized within the basal ganglia^29,40^, and transmitted through the brainstem^41–43^ to the spinal cord, where movement signals are converted into synergistic muscle contractions^30^. Degeneration of the basal ganglia dopamine system in PD interferes with the execution of movement sequences. Kinematic analysis in laboratory settings during motor learning sequencing tasks has shown that PD patients after levodopa medication are able to better perform sequential movements^44,45^. When directly recorded at the muscle level, reduced muscle synergy has been observed in PD patients^46,47^. In agreement with these observations, our study showed a reduction of movements with disease severity and an improvement with levodopa medications. This is likely a different mechanism than that seen in the reorganization of synergistic movements observed in stroke patients^48,49^. Although many studies support the disruption of sequences of movements in PD, they are constrained by episodic observations in laboratory settings. The present study bridges the gap between clinic/laboratory and at-home studies by showing that the same methods can be applied continuously during daily fluctuations.

In summary, we have shown how the stationary representation of movement syllables captures the overall quality of motor behavior. The proposed method differentiates motor impairment accompanying various PD related states both in clinical settings when scripted activities are performed, and in unconstrained everyday life. The proposed model was explicitly tested on movement differences associated with PD but we see no reasons why it should not be generalizable to detecting other neurological states with characteristic movement signatures.

## Supplementary information

Supplementary Information.
