# Supplementary material for: Using an unbiased symbolic movement representation to characterize Parkinson’s disease states
Source: Sci Rep. 2020 Apr 30;10:7377. doi: 10.1038/s41598-020-64181-3 (PMC7193555; doi:10.1038/s41598-020-64181-3)
Supplement: Supplementary file 1 — Supplementary Information. [file 41598_2020_64181_MOESM1_ESM.docx]

**Supplementary Material**

**Title:** Using an unbiased symbolic movement representation to characterize Parkinson’s disease states

**Authors:** Avner Abrami, Stephen Heisig, Vesper Ramos, Kevin C. Thomas, Bryan K. Ho, Vittorio Caggiano

SUPPLEMENTAR FIGURES:


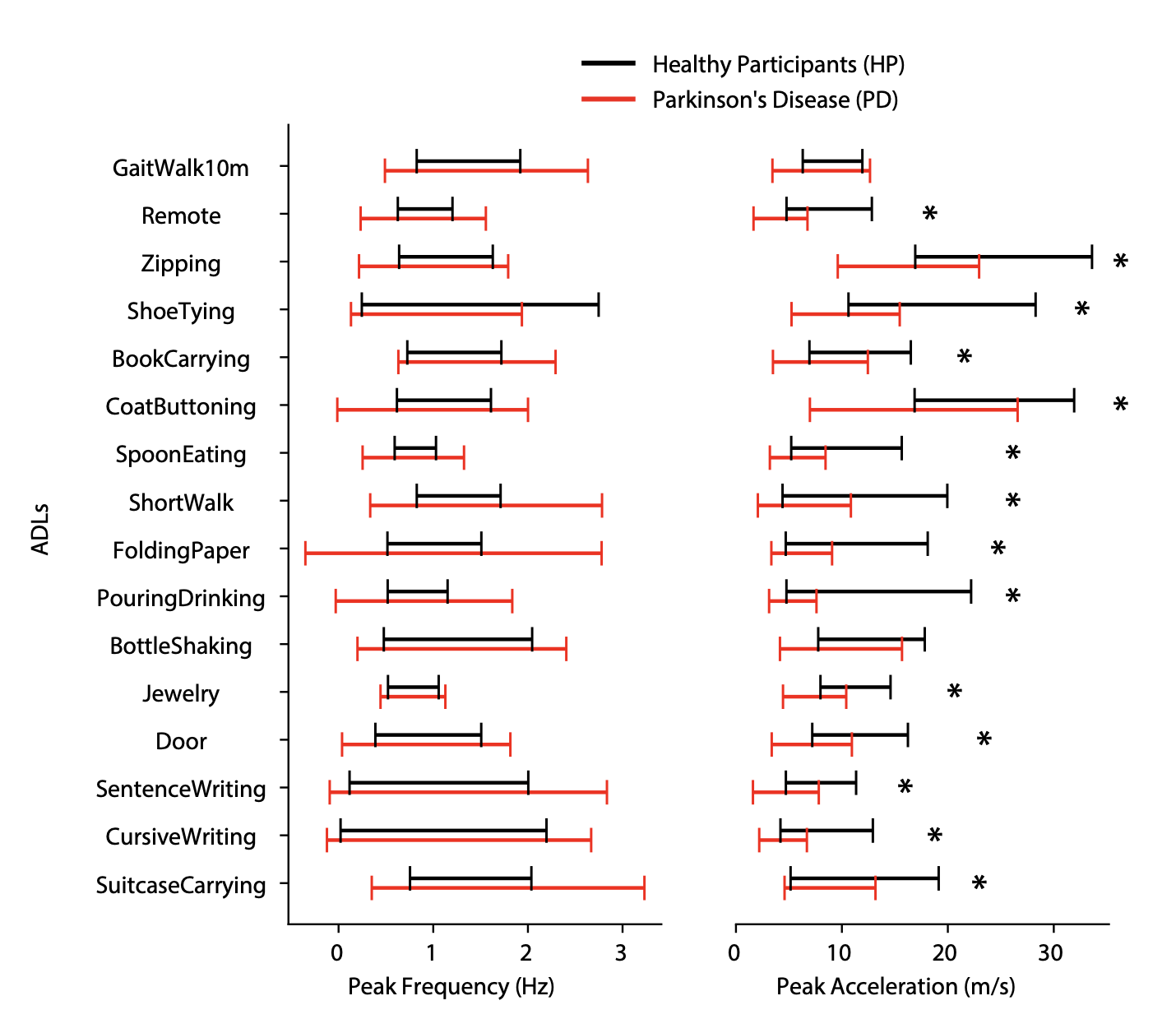


**Supplementary Figure 1 –** Distribution of magnitude peak frequency (left) and peak acceleration (right) of healthy participants (HP– black) and Parkinson’s Disease patients (PD – red) performing scripted Activities of the Daily Living (ADLs).

**Supplementary Figure 2 – Effects of the Vocabulary Size on the Sum of Squared Error (SSE).** In the figure inset a magnification of the vocabulary size between 2 and 45 classes shows how from a size of 24 there is a monotonically descending trend.

**Supplementary Figure 3 – Acceleration templates for each syllable.** Each plot shows the x, y, z accelerometer information for each syllable (index starts from 0).


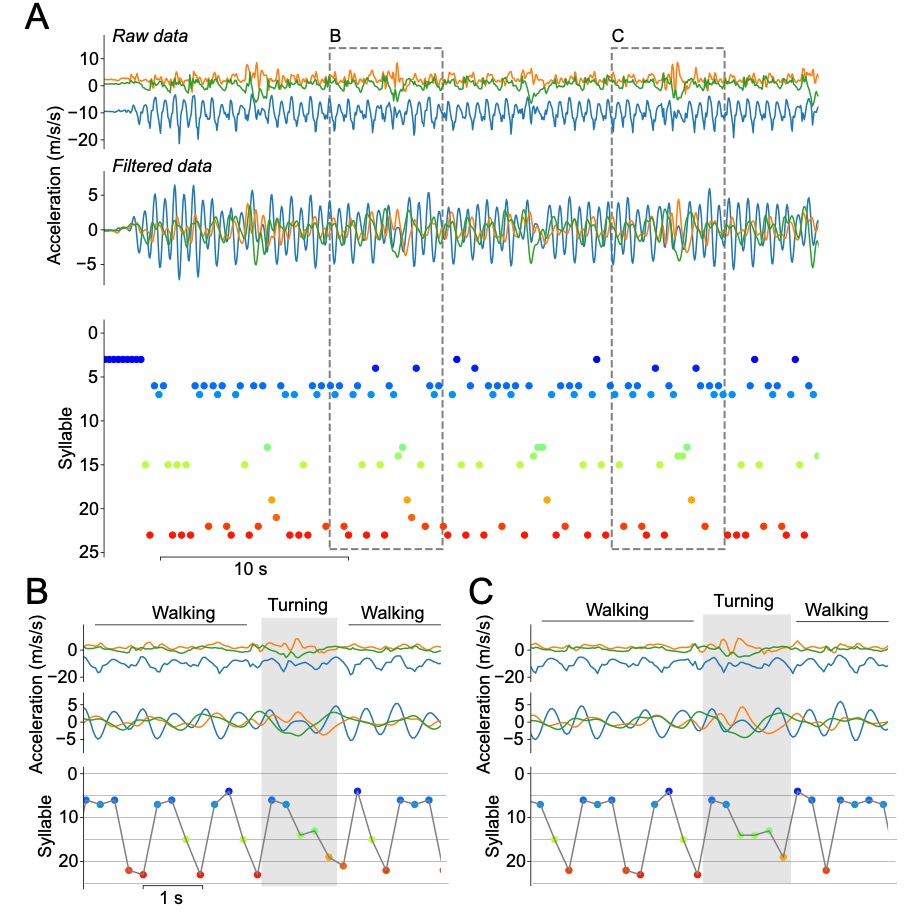


**Supplementary Figure 4 –** Example of raw and filtered accelerometer data, and syllable sequence during walking and turning. **A** – Subject starting from a still position, starts a series of walking and turning bouts. Raw (upper row) and filtered (middle row) extracted from the wrist accelerometer (see Figure 1) during this task and associated syllable sequence (lowest row) are shown. Note that the index of the syllable starts from 0. **B and C** – Magnification of the walking sequence around turning movements highligths how during arm swing movements the sequence of actions is consistenly transitioning between syllables 4 – 6 – 7 - 15 – 23 while during turing the sequence is through states 13 – 14 - 19 - 21.

**
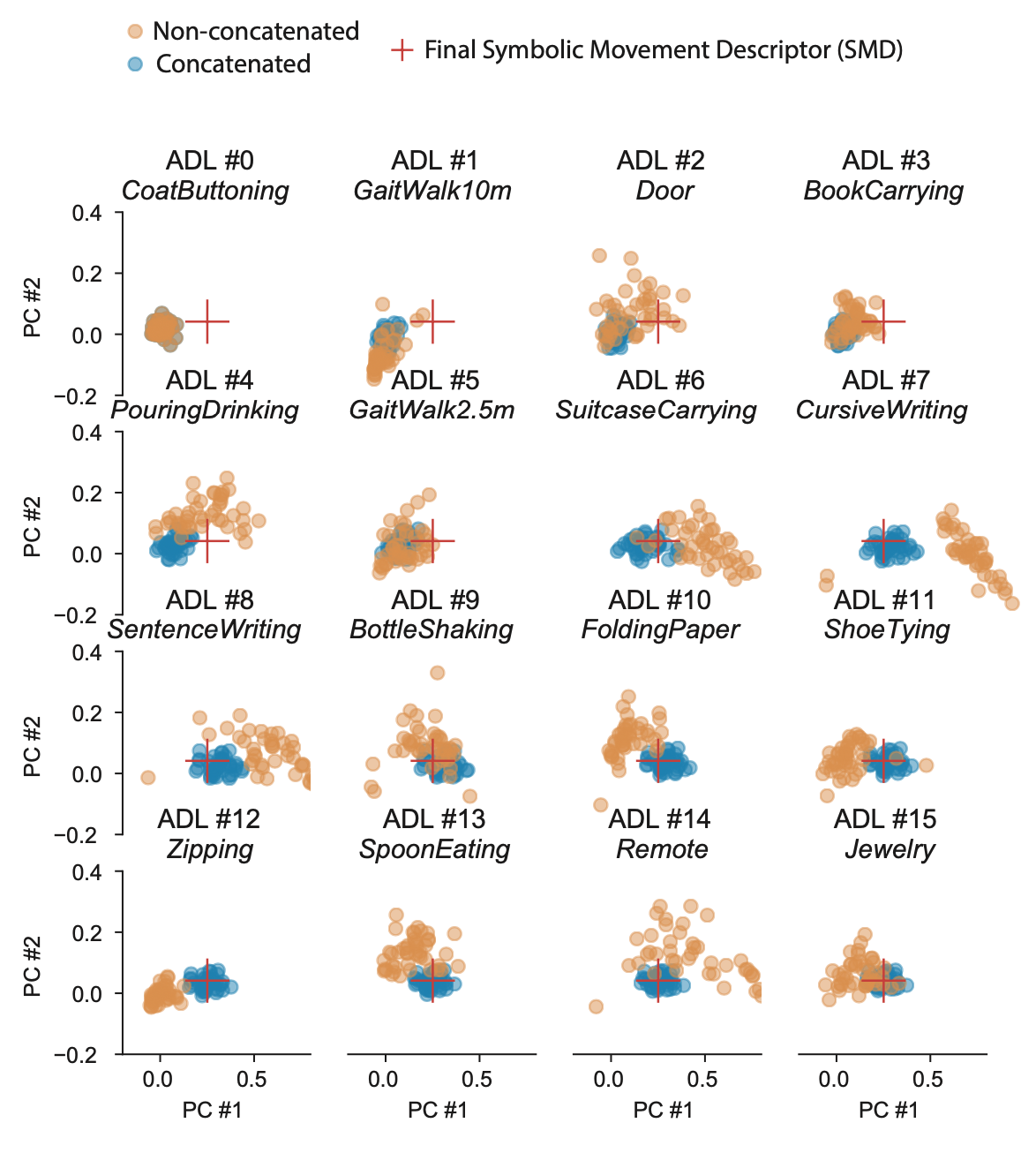
**

**Supplementary Figure 5 –** Projection of the Symbolic Movement Representation on the first two principal component axis for each ADL. Orange circles represent the activities of each subject in each ADLs when actions are considered in isolation. Blue circles indicate the projections when actions are concatenated. Red Cross represents the center of the projections of the distribution that concatenates all actions.


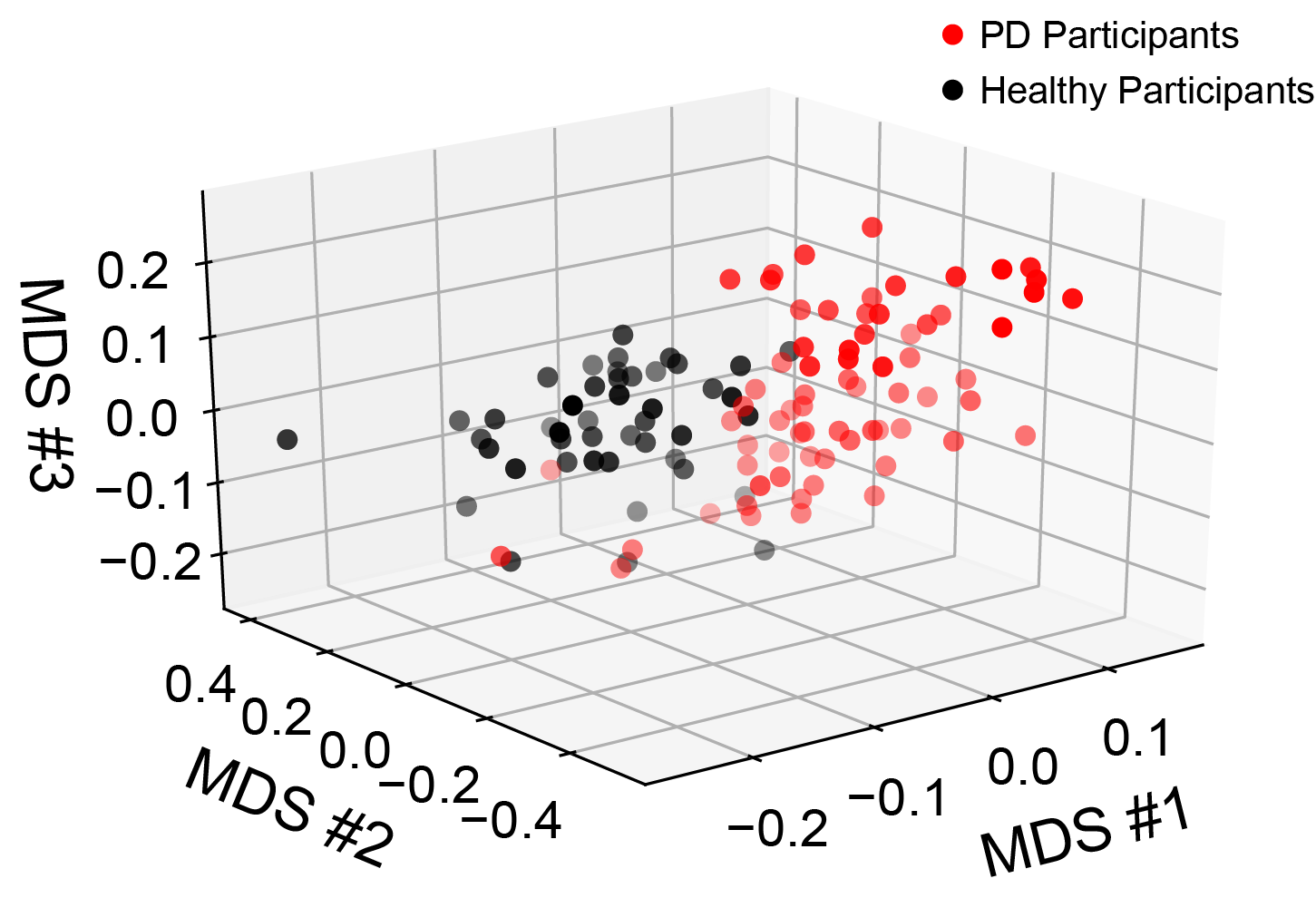


**Supplementary Figure 6 – MDS projection of all healthy participants (Black) against PD participants (Red).** Scatter plot of the whole population of healthy and PD participants in the clinic studies. See also Figure 3 and 4.


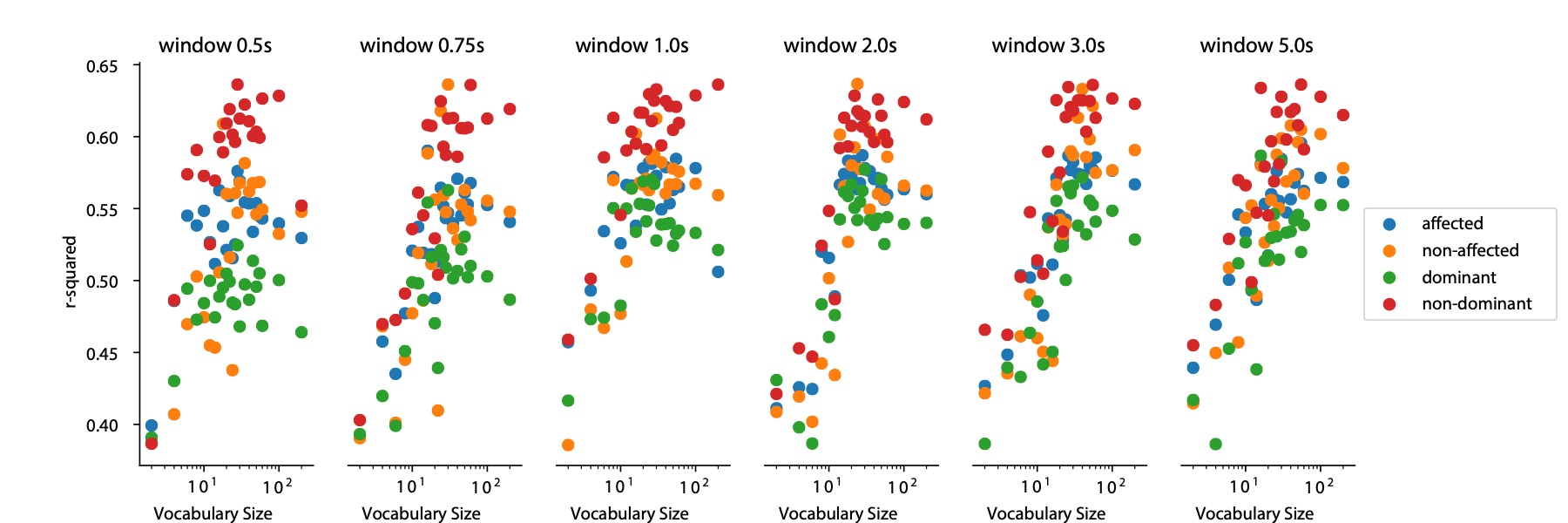


**Supplementary Figure 7** – **Effect of the window size and vocabulary size on correlation with disease severity.** Analysis of the correlation with the neurologist assesment as function of key paramenters used in the analysis: vocabulary size and window size. Results show a consistent and reliable effect with a vocabulary size grater than 24 (see also Supplementary Figure 2).


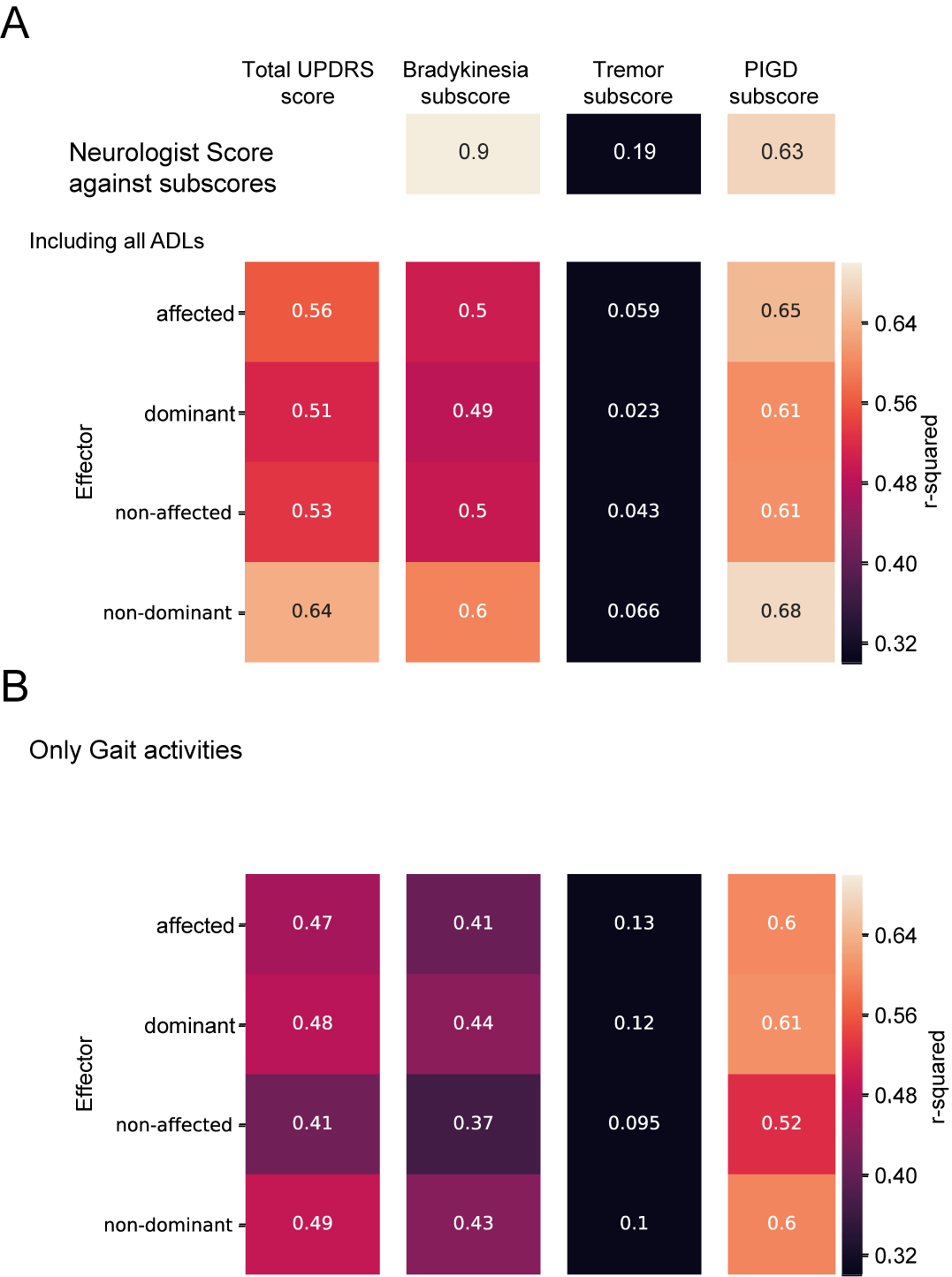


**Supplementary Figure 8** **– Effect of the position of the sensor and subscores. A** – On the top, linear regression of the Bradykinesia, Tremor and PIGD subscores (see Methods for the definition of subscores) against Total UPDRS-III score. On the bottom, effect of the position of the sensor on the wrist of the affected, non-affected, dominant, non-dominant hand with respect to the Total UPDRS-III score, and subscores. **B** – Results of the regression model on the effectors and subscores when only gait activities are considered.
